# Use of Artificial Intelligence to Improve the Quality Control of Gastrointestinal Endoscopy

**DOI:** 10.3389/fmed.2021.709347

**Published:** 2021-07-22

**Authors:** Ya-qi Song, Xin-li Mao, Xian-bin Zhou, Sai-qin He, Ya-hong Chen, Li-hui Zhang, Shi-wen Xu, Ling-ling Yan, Shen-ping Tang, Li-ping Ye, Shao-wei Li

**Affiliations:** ^1^Taizhou Hospital, Zhejiang University, Linhai, China; ^2^Key Laboratory of Minimally Invasive Techniques and Rapid Rehabilitation of Digestive System Tumor of Zhejiang Province, Taizhou Hospital Affiliated to Wenzhou Medical University, Linhai, China; ^3^Department of Gastroenterology, Taizhou Hospital of Zhejiang Province Affiliated to Wenzhou Medical University, Linhai, China; ^4^Health Management Center, Taizhou Hospital of Zhejiang Province Affiliated to Wenzhou Medical University, Linhai, China; ^5^Department of Gastroenterology, Renmin Hospital of Wuhan University, Wuhan, China; ^6^Taizhou Hospital of Zhejiang Province Affiliated to Wenzhou Medical University, Linhai, China; ^7^Institute of Digestive Disease, Taizhou Hospital of Zhejiang Province Affiliated to Wenzhou Medical University, Linhai, China

**Keywords:** application, artificial intelligence, quality control, improving, gastrointestinal endoscopy

## Abstract

With the rapid development of science and technology, artificial intelligence (AI) systems are becoming ubiquitous, and their utility in gastroenteroscopy is beginning to be recognized. Digestive endoscopy is a conventional and reliable method of examining and diagnosing digestive tract diseases. However, with the increase in the number and types of endoscopy, problems such as a lack of skilled endoscopists and difference in the professional skill of doctors with different degrees of experience have become increasingly apparent. Most studies thus far have focused on using computers to detect and diagnose lesions, but improving the quality of endoscopic examination process itself is the basis for improving the detection rate and correctly diagnosing diseases. In the present study, we mainly reviewed the role of AI in monitoring systems, mainly through the endoscopic examination time, reducing the blind spot rate, improving the success rate for detecting high-risk lesions, evaluating intestinal preparation, increasing the detection rate of polyps, automatically collecting maps and writing reports. AI can even perform quality control evaluations for endoscopists, improve the detection rate of endoscopic lesions and reduce the burden on endoscopists.

## Introduction

Artificial intelligence (AI) is a new and powerful technology. In contrast to machines, the human brain may make mistakes in long-term work due to fatigue and stress, among other distractions; AI technology can therefore compensate for the limited capabilities of humans. Over the past few decades, AI has received increasing attention in the field of biomedicine. A multidisciplinary meeting was held on September 28, 2019, where academic, industry and regulatory experts from different fields discussed technological advances in AI in gastroenterology research and agreed that AI will transform the field of gastroenterology, especially in endoscopy and image interpretation (1). In fact, there are many cases of missed lesion detection due to low-quality endoscopy, which can be greatly reduced with the help of AI.

Thus far, AI has mainly been applied to the field of endoscopy in two aspects: computer-aided detection (CADe) and computer-aided diagnosis (CADx) (2). Although many of the advantageous features of AI seem promising for routine endoscopy, endoscopy still depends heavily on the technical skills of the endoscopist. Improving the quality of endoscopy is thus needed to improve the detection rate and ensure the correct diagnosis of diseases.

**Figure 1:**
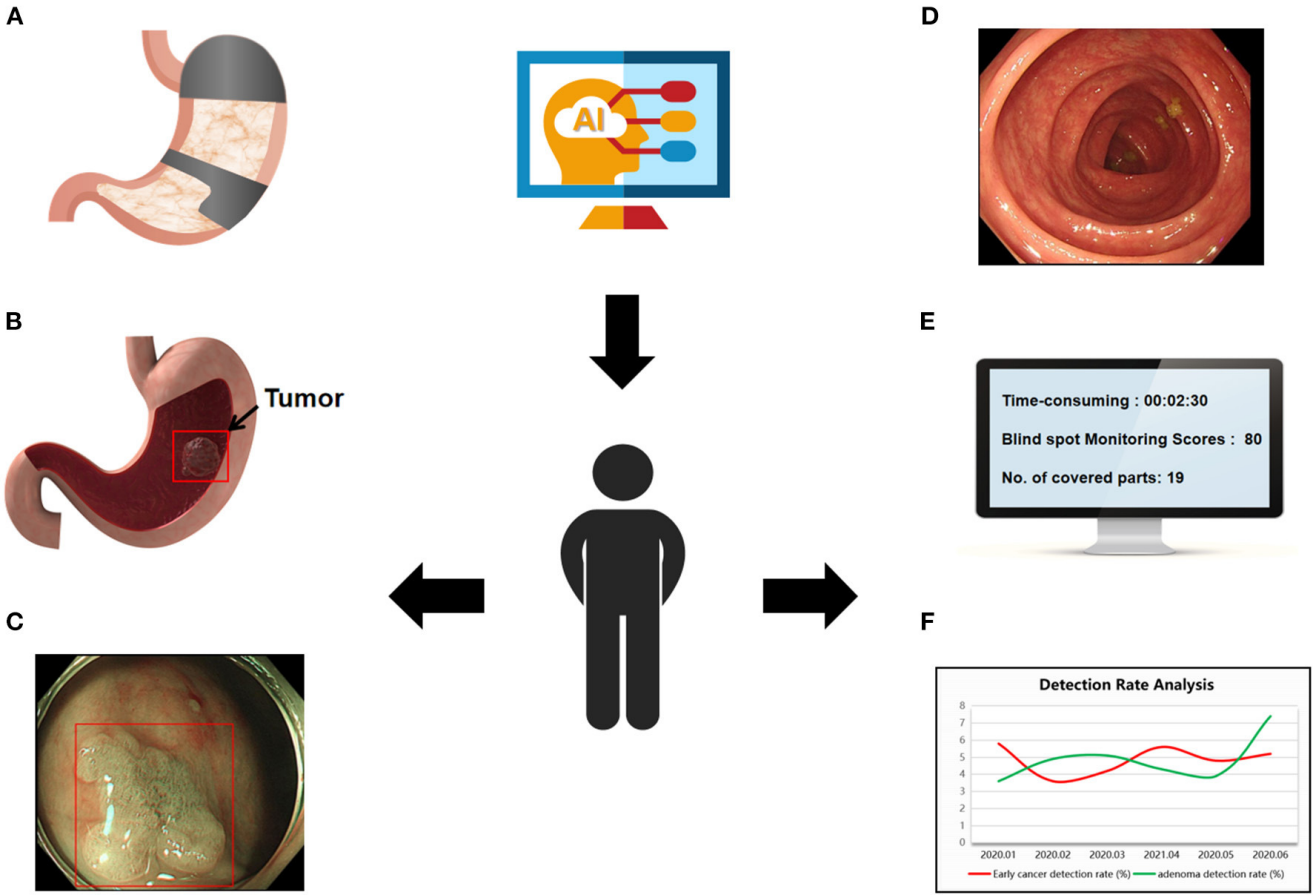
Use of AI in gastrointestinal endoscopy. **(A)** Display the examined site, reduce the blind spot rate of endoscopy. **(B,C)** Determine the depth and boundary of gastric cancer invasion. **(D)** Automated bowel scoring. **(E)** Real-time recording of operation time, inspected parts, and scores. **(F)** Trend analysis of endoscopy quality.

In this review, we summarize the literature on AI in gastrointestinal endoscopy, focusing on the role of AI in monitoring (Figure 1)—mainly in monitoring the endoscopy time, reducing endoscopy blindness, improving the success rate of high-risk lesion detection, evaluating bowel preparation, increasing polyp detection rate and automatically taking pictures and writing reports, with the goal of improving the quality of daily endoscopy and making AI a powerful assistant to endoscopists in the detection and diagnosis of disease.

### Terms Related to AI

In recent years, the proliferation of AI-based applications has rapidly changed the way we work and live. AI refers to the ability of a machine or computer to learn and solve problems by imitating the human mind with human-like cognition and task execution (3).

Machine learning (ML) and deep learning (DL) can be considered subsets of AI. Machine learning is a fundamental concept in AI, which can be described as the study of computer algorithms that are automatically improved through training and practice over time (4). This approach requires human input of meaningful image features into a trainable prediction algorithm, such as a classifier (5). Deep learning (DL) is a transformative machine-learning technique that enables transfer learning, where parameters in each layer are changed based on representations in previous layers, and can be effectively applied even when the new task has a limited training data set (6).

Artificial neural networks (ANNs) are supervised models that are very similar to the organization of the human central nervous system. Convolutional neural networks (CNNs) are an even more advanced digital DL technique widely used in image and pattern recognition. CNNs are similar to the human brain in their approach to thinking and use large image data sets for learning. Usually, the data set is divided randomly, and a subset is reserved for cross-validation (7).

### Application of AI in the Gastrointestinal Tract

#### Identifying Anatomy

For upper gastrointestinal endoscopy, the European Society of Gastrointestinal Endoscopy (EGSE) has proposed the collection of images of eight specific upper gastrointestinal (UGI) landmarks (8), and several similar classification methods have been developed. AI has proven useful for identifying and labeling anatomical sites of the upper digestive tract. Takiyama et al. designed a CNN to identify the anatomical location of esophagus gastroduodenoscopy (EGD) images. They collected 27,335 EGD images for training and divided them into four main anatomical parts (larynx, esophagus, stomach and duodenum) with three sub-classifications of the stomach (upper, middle and lower). The accuracy rate was found to be 97%, but the clinical application was limited (9). The Wisense AI system designed by Wu et al. classified 26 EGD sites and monitored blind spots in real time through reinforcement learning, achieving an accuracy rate of 90.02% and making significant progress in real time (10, 11). Seong Ji Choi et al. developed an AI-driven quality control system for EGD using CNNs with 2,599 retrospectively collected and labeled images obtained from 250 EGD surgeries. The EGD images were classified into 8 locations using the developed model, with an accuracy of 97.58% and sensitivity of 97.42% (12).

In the lower digestive tract, an AI system can automatically identify the cecum and monitor the speed of endoscopic withdrawal. Samarasena et al. developed a CNN that can automatically detect equipment during endoscopy, such as snares, forceps, argon plasma coagulation catheter, endoscopic auxiliary equipment, anatomical cap, clamps, dilating balloons, rings and injection needles. The accuracy, sensitivity and specificity of these devices detected by the CNN were 0.97, 0.95 and 0.97, respectively (13). Based on the function of the recognition device, the AI system can further help accurately measure the size of the polyp and aid the endoscopist in quickly determining whether to leave it in place or remove and discard it. Karnes et al. developed a CNN to automatically identify the cecum (13), and the ENDOANGEL is further able to monitor the exit speed, colonoscopy intubation and exit timing and alert the endoscopic surgeon to blind spots caused by endoscopic sliding (14). Identifying the anatomical parts of the digestive tract and accurately classifying them can help inexperienced endoscopists correctly locate the examination site as well as reduce the blind spot rate.

#### Reducing the Blind Spot Rate of Endoscopy

Gastric and esophageal cancers are common cancers of the digestive tract but can easily be missed during endoscopy, especially in countries where the incidence of the disease is low and training is limited. The 5-year survival rate of gastric cancer is highly correlated with the stage of gastric cancer at the time of the diagnosis, so it is very important to improve the detection rate of early gastrointestinal (GI) cancer. Some blind spots in the gastric mucosa, such as the sinus and the small curvature of the fundus, may be hidden from the endoscopist, depending to a large extent on the competence of the endoscopist.

To reduce the blind spot rate of EGD surgery, Wu et al. built a real-time quality improvement system known as WISENSE. Through training on 34,513 stomach images, blind spots were detected in real EGD videos with an accuracy of 90.40%. In a single-center randomized controlled trial, the blind spot rates of the WISENSE group and the control group were 5.86 and 22.46%, respectively, indicating a significant reduction in the blind spot rate with the WISENSE. In addition, the WISENSE can automatically create photo files, thus improving the quality of daily endoscopy (10).

In a prospective, single-blind, randomized controlled trial, 437 patients were randomly assigned to unsedated ultrathin transoral endoscopy (U-TOE), unsedated conventional Esophagogastroduodenoscopy (c-EGD) or sedated c-EGD, and each group was divided into two subgroups according to the presence or absence of assistance from an AI system. Among all groups, the blind spot rate in the AI-assisted group was 3.42%, which was much lower than that in the control group (22.46%), and the addition of AI had the greatest effect on the sedated c-EGD group (11).

#### Guided Biopsy

Squamous cell carcinoma of the pharynx and esophagus is a common disease, and one randomized controlled study indicated that the specificity of esophageal carcinoma was no more than 42.1%, while the sensitivity was only 53% for inexperienced physicians (15, 16). Seattle protocols and evolving imaging technologies can assist in the diagnosis, but some issues remain, such as the need for expert handling, a low sensitivity and sampling errors (17, 18).

The American Society of Gastrointestinal Endoscopy recognizes the use of advanced imaging technology to switch from a random biopsy to a targeted biopsy under certain circumstances. Imaging techniques with targeted biopsies for detecting high-grade dysplasia (HGD) or early esophageal adenocarcinoma (EAC) achieve ≥90% sensitivity, negative predictive values of ≥98% and sufficiently high specificity (80%) to reduce the number of biopsies (19). However, this requires a long learning period, and only experienced endoscopists can reach this level.

An AI system can help endoscopists switch from a random biopsy to a targeted biopsy and improve the detection rate of endoscopic lesions without the need for complicated training procedures. To improve the detection of early esophageal tumors, de Groof et al. validated a DL-based CADe system using five independent datasets. The CAD system classified images as neoplasms or non-dysplastic BE with 89% accuracy, 90% sensitivity and 88% specificity. In addition, in 2 other validation datasets, the system accurately located the best location for biopsy in 97 and 92% of cases (20). The CNN constructed by Shichijo et al. was used for *Helicobacter pylori* detection by classifying the anatomical parts of the stomach (21, 22). The sensitivity, specificity and accuracy were increased compared with endoscopists, improving the choice of the biopsy location (21, 23).

Traditionally, a biopsy has been used to assess the nature of lesions. However, CADx systems can help predict histology, even in the absence of biopsy. Endocytoscopy is a contact microscopy procedure that allows for the real-time assessment of cell, tissue and blood vessel atypia *in vivo*. EndoBRAIN, a combination of endocytoscopy and narrow-band imaging (NBI), is a platform for performing automated optical biopsies that was validated and evaluated on 100 images of colorectal lesions resected endoscopically and subjected to pathology; the EndoBRAIN system shows an accuracy of 90% (24). Using laser-induced autofluorescence spectroscopy, which combines optical fibers into standard biopsy forceps and triggers upon contact, the WAVSTAT4 system provides a real-time, *in vivo* automatic optical biopsy of colon polyps. When validated prospectively in 137 polyps, the accuracy of the WAVSTAT4 system was found to be 85% (25). The use of the CADx systems can help reduce uneven level in the levels of observers, thereby improving standardization and enabling wider adoption by less-experienced endoscopists (26).

#### Determining the Depth and Boundary of Gastric Cancer Invasion

Gastric cancer is a common cancer of the digestive tract, and early cancer recognition tests are particularly important. However, an early endoscopic diagnosis is difficult, as most early gastric cancers show only a slight depression or bulge with a faint red color. Predicting the depth of infiltration of the gastric wall is a difficult task, and making an optical diagnosis using image enhancement techniques, flexible spectral imaging color enhancement (FICE) or blue-laser imaging (BLI) has proven useful, provided that the endoscopist has a great deal of expertise. AI helps solve the issue of endoscopists having too little experience (27).

To investigate the depth of esophageal squamous cell carcinoma (ESCC) invasion, two Japanese research groups developed and trained the CADX system separately. The sensitivity and accuracy of the system studied by Nakagawa et al. to distinguish pathological mucosal and submucosal microinvasive carcinoma from submucosal deep invasive carcinoma were 90.1 and 91.0%, respectively, and the specificity was 95.8%. The system was compared to the findings of 16 experienced endoscopic specialists, and its performance was shown to be comparable (28). Tokai et al.'s CADX system detected 95.5% of ESCCs (279/291) in the test images within 10 s and correctly estimated the depth of infiltration with a sensitivity of 84.1% and an accuracy of 80.9%, which was better than the accuracy of 12 of the 13 endoscopic experts (29). Kubota et al. developed a CADx model for diagnosing the depth of early gastric cancer invasion on gastroscopic images. About 800 images were used for computer learning, and the overall accuracy rate was 64.7%. The diagnostic accuracy rates of the T1, T2, T3, and T4 stages were 77.2, 49.1, 51.0, and 55.3%, respectively (30). Zhu et al. designed a CNN algorithm using 790 endoscopic images for training and another 203 for verification to assess the depth of invasion of gastric cancer. The accuracy of the system was 89.2%, the sensitivity was 74.5%, and the specificity was 95.6% (31).

Using magnified NBI images, Kanesaka et al. developed a CADe tool that can be used for detection, in addition to depicting the border between cancerous and non-cancerous gastric lesions, with 96.3% accuracy, 96.7% sensitivity and 95% specificity (32). Miyaki et al. developed a support vector machine (SVM)-based analysis system for the quantitative identification of gastric cancer together with BLI endoscopy. The training set was made using 587 images of gastric cancer and 503 images of surrounding normal tissue, and the validation set comes from 100 EGC images of 95 patients. These images were all examined by BLI magnification using the laser endoscopy system. The results showed that the average SVM output value of cancerous lesions was 0.846 ± 0.220, that of red lesions was 0.381 ± 0.349, and that of the surrounding tissue was 0.219 ± 0.277. The SVM output value of cancerous lesions was significantly greater than that of the red lesions or surrounding tissue. The mean output of undifferentiated cancer was greater than that of differentiated cancer (33).

#### Identifying and Characterizing Colorectal Lesions

Polyp size measurements are important for the effective diagnosis, treatment and establishment of monitoring intervals. Wang et al. developed an algorithm that uses edge cross-sectional visual features and rule-based classifiers to detect the edges of polyps and track the edges of the detected polyps. The program correctly detected 42 of 43 polyp shots (97.7%) from 53 videos randomly selected by 2 different endoscope processors. The system can help endoscopists discover more polyps in clinical practice (34). Requa et al. (35) developed a CNN to estimate the size of polyps on colonoscopy. This system can run during real-time colonoscopy and divide polyps into 3 size-based groups of ≤5, 6–9, and ≥10 mm, with the final model showing an accuracy of 0.97, 0.97 and 0.98, respectively. Byrne et al. also described a real-time evaluable deep neural network (DNN) model for polyp detection with an accuracy, sensitivity, specificity, negative predictive value and positive predictive value of 94.0, 98.0, 83.0, 97.0, and 90.0% for adenoma differentiation (36).

Ito et al. developed an endoscopic CNN to distinguish the depth of invasion of malignant colon polyps. The sensitivity, specificity and accuracy of the system for the diagnosis of deep invasion (cT1b) were 67.5, 89.0, and 81.2%, respectively. The use of a computer-assisted endoscopic diagnostic support system allows for a quantitative diagnosis to be made without relying on the skills and experience of the endoscopist (37).

The use of AI systems as clinical adjunct support devices allows for more extensive use of “leave in place” and “remove and discard” strategies for managing small colorectal polyps. Chen et al. developed a CADx system with a DNN-CAD for the identification of neoplastic or proliferative colorectal polyps smaller than 5 mm in size. The training set consisted of 1,476 images of neoplastic polyps and 681 images of proliferative polyps, and the test set consisted of 96 images of proliferative polyps and 188 images of small neoplastic polyps. The system achieved 96.3% sensitivity, 78.1% specificity and 90.1% accuracy in differentiating tumors from proliferative polyps. The DNN-CAD system was able to classify polyps more quickly than either specialists or non-specialists (38).

#### Automated Assessment of Bowel Cleansing

The adenoma detection rate (ADR) is widely accepted measure of the quality of colonoscopy, defined as the percentage of patients who have at least one adenoma detected during colonoscopy performed by an endoscopist. The ADR is negatively correlated with the risk of interstage colorectal cancer, and there is a strong positive correlation between the quality of bowel preparation and the colon ADR. A variety of tools have been developed to assess intestinal readiness, such as the Boston Bowel Preparedness Scale (BBPS) and the Ottawa Bowel Preparedness Scale, but subjective biases and differences also exist among endoscopic physicians. The bowel preparation scale is another indicator that can be automatically evaluated by AI, with good results achieved. A proof-of-concept study using AI models to evaluate quality measures such as the mucosal surface area and bowel readiness score examined the sufficiency of colonic dilation and clarity of endoscopic views (39). Another study used a deep CNN to develop a novel system called the ENDOANGEL to evaluate bowel preparation. The ENDOANGEL ultimately achieved 93.33% accuracy in 120 images and 89.04% in 20 real-time inspection videos, which is higher than the accuracy rate of the endoscopists consulted for the study. The accuracy rate, in 100 images with bubbles, also reached 80.00% (40).

The software program developed by Philip et al. to provide feedback on the quality of colonoscopy works in three ways: measuring the sharpness of the image from the video in real time, assessing the speed of exit and determining the degree of bowel preparation. Fourteen screening colonoscopy videos were analyzed, and the results were compared with those of three gastroenterology experts. For all of colonoscopy video samples, the median quality ratings for the automated system and reviewers were 3.45 and 3.00, respectively. In addition, the better the endoscopist withdrawal speed score, the higher the automated overall quality score (41).

In a recent study, Gong et al. (42) established a real-time intelligent digestive endoscopy quality control system capable of retrospectively analyzing endoscopy data and helping endoscopists understand inspection-related indicators, such as the inspection time and blindness rate, ADR and bowel preparation success rate. The complaint report can be generated automatically, and these data can further analyze the changing trend of the detection rate of colonoscopy adenoma and precancerous lesions, so as to help endoscopists to analyze their own shortcomings and make improvements.

#### Identifying and Characterizing UGI Tract Lesions

Advanced esophageal and gastric cancer often have a poor prognosis, so early upper gastrointestinal (UGI) endoscopic detection is especially important. In European community, the missed diagnosis rate for UGI cancers has been reported to range from 5 to 11%, while the rate for Barrett's early stage tumors has been reported to be as high as 40% (43). AI systems could help endoscopists detect upper digestive tract tumors and improve the detection rate. However, these systems are still experimental in design and there is still uncertainty about their clinical applicability.

In order to explore the diagnostic performance of AI in detecting and characterizing UGI tract lesions, Julia Arribas et al. searched relevant databases before July 2020 and analyzed and evaluated the comprehensive diagnostic accuracy, sensitivity and specificity of AI. According to the meta-analysis, the AI system showed high accuracy in detecting UGI tumor lesions, and its high performance covered all ranges of UGI tumor lesions [including esophageal squamous cell neoplasia (ESCN), Barrett's esophagus-related neoplasia (BERN), and gastric adenocarcinoma (GCA)]. The sensitivity of AI to detect UGI tumors was 90%, the specificity was 89%, and the total AUC was 0.95 (CI 0.93–0.97) (43).

Leonardo Frazzoni et al. evaluated the accuracy of endoscopic physicians in identifying UGI tumors using the AI validation research framework, with an AUC of 0.90 for ESCN (95%CI 0.88–0.92) and 0.86 for Bern (95%CI 0.84–0.88). The results showed that the accuracy of endoscopists in identifying UGI tumors was not particularly good, and suggested that AI validation studies could be used as a framework for evaluating endoscopists' capabilities in the future (44).

**Figure 2:**
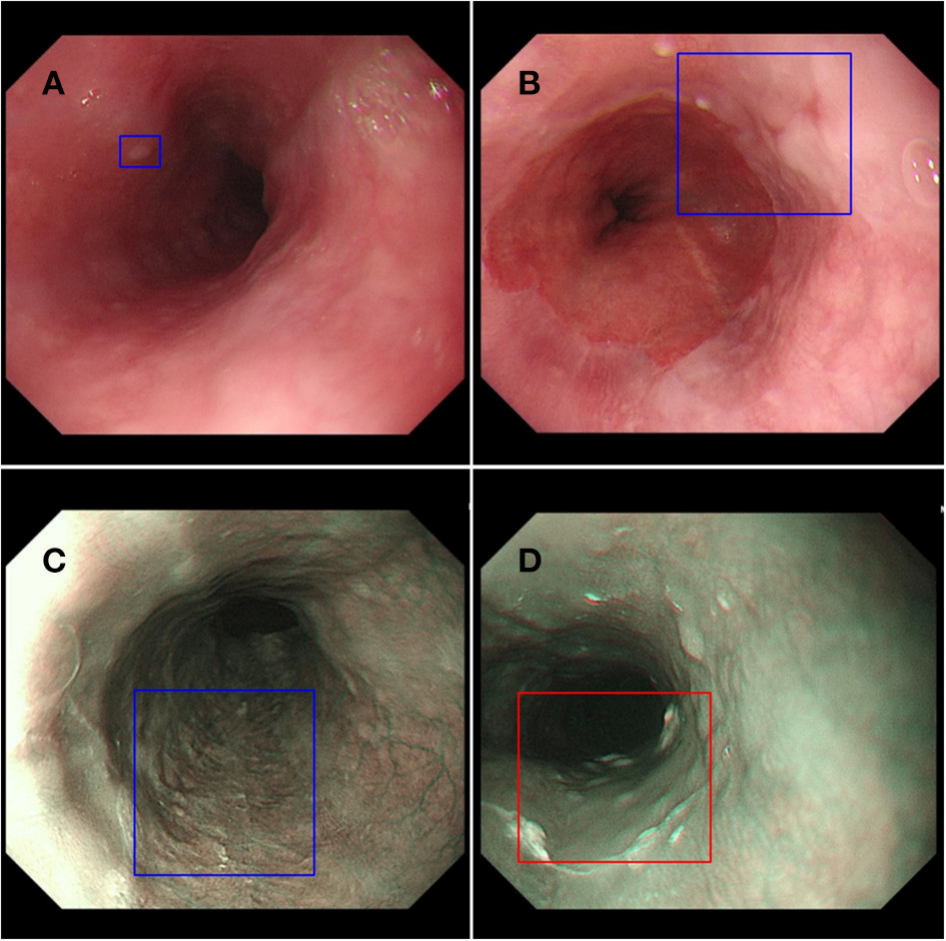
ENDOANGEL monitors esophageal lesions. **(A,B)** Low-risk lesion of the esophagus in the endoscopic white light mode. **(C)** Low-risk lesion of the esophagus in the endoscopic NBI mode. **(D)** High-risk lesion of the esophagus in the endoscopic NBI mode.

In order to explore the clinical applicability of AI in improving the detection rate of early esophageal cancer, we designed a prospective randomized, single-blind, parallel controlled experiment to evaluate the effectiveness of AI system ENDOANGEL in improving the detection of high-risk lesions in the esophagus (Figure 2). ENDOANGEL is an AI model based on a deep learning algorithm that recognizes and prompts high and low-risk esophageal lesions under NM-NBI. It outlines the range of suspicious lesions in the form of a prompt box and gives a risk rating. We hope ENDOANGEL can increase the detection rate of high-risk esophageal lesions by electronic esophageal gastroscopy. At present, this clinical study is in progress. In the early stage, we used a large number of gastroscopy videos of high-risk esophageal lesions to train the model. In the pre-experimental stage, it was found that the model had a problem of misjudgment in the cardia, that is, the dentate line was mistaken for the lesion is framed. In order to reduce the misjudgment rate, we have further trained the model, and this problem has been well-improved after learning. At the same time, as in other studies, this model occasionally mistakes bubbles and mucus for lesions. For now, AI is not perfect, but just like the problem encountered in this experiment, through deeper learning and continuous training, the error rate will gradually decrease to ensure a high correct detection rate.

## Conclusion

In gastrointestinal endoscopy, computer-aided detection and diagnosis have made some progress. Table 1 summarizes the key research on the diverse functions of AI in the application of gastrointestinal endoscopy. At the present, CADe and CADx have helped endoscopists improve detection rates for many diseases, but there are still many limitations to its implementation and use. First, research on AI is still in the early stages, and static images are usually used to verify computer-aided design models. Most of these studies are retrospective and lack of prospective experiments. Second, computer-aided endoscopy systems are often plagued by false positives, such as air bubbles, mucus and feces and exposure. Third, most of these systems are developed and designed by a single institution for use in certain patient groups, so their expansion to other populations may be difficult. However, it is undeniable that the prospects for the auxiliary application of AI in GI endoscopy are bright. In remote or backward areas, endoscopic technology is difficult to be guaranteed, and the skills of endoscopists grow slowly. Computer-aided examination can help solve the problems of high rate of missed diagnosis and false diagnosis.

**Table 1:** The role of AI in quality control of gastroenteroscopy.

**Functional classification**	**Areas of assistance**	**Specific application**	**References**
Identifying anatomy	Identify the upper digestive tract	Divided into 8 or 26 parts	Choi et al. (12)
			Wu et al. (10, 11)
	Identify the lower digestive tract	Measure polyp size	Abadir et al. (13)
		Monitor the speed of mirror withdrawal	Hassan et al. (14)
Reducing the blind spot rate of endoscopy	Reduce the blind spot rate of gastroscopy	Real-time monitoring and monitoring of blind spots	Wu et al. (10)
		AI-assisted sedation of c-EGD was most effective in reducing the rate of blind spots	Chen et al. (11)
Guided biopsy	Barrett esophagus positioning biopsy	Distinguish neoplastic or hyperplastic	Sharma et al. (19)
	*Helicobacter pylori* detection	Locate the anatomical site of the stomach	Shichijo et al. (21), Gulati et al. (22)
	Optical biopsies of endoscopic cells	Detect colon lesions	Misawa et al. (24)
	Fiber optic positioning biopsy	Intestinal polyp nature determination	Rath et al. (25)
Determining the depth and boundary of gastric cancer invasion	Differentiate the depth of infiltration of esophageal squamous cell carcinoma	Distinguish between microinvasive carcinoma and deep invasive carcinoma	Nakagawa et al. (28)
	Diagnose the early gastric cancer infiltration depth	Use the invasion depth of endoscope images to determine the wall of the stomach	Kubota et al. (30), Zhu et al. (31)
	Delineate the gastric cancer boundary	Use enlarged NBI images to delineate the relationship between cancerous and non-cancerous gastric lesions	Kanesaka et al. (32)
	Quantitative identification of gastric cancer	Based on a support vector machine analysis of different output values, quantitatively identify gastric cancer	Miyaki et al. (33)
Identifying and characterizing colorectal lesions	Identify polyp size	Degree of recognizing different polyp sizes (≤5 mm, 6–9 mm and ≥10 mm)	Requa et al. (35)
	Infiltrating depth difference between malignant polyps	CNN system for diagnosing a CT1B polyp	Ito et al. (37)
Automated assessment of bowel cleansing	Assess bowel preparation for examinations	The accuracy of ENDOANGEL was higher than that of professional endoscopists.	Zhou et al. (40)
	The sharpness of the video image, speed of exit and level of intestinal preparation were measured	The automatic system has high accuracy in scoring	Filip et al. (41)

It's worth noting that AI systems cannot completely replace endoscopes, even with further improvements in the future. Most current AI systems are tested for specific diseases in specific areas. In the future, we expect that AI can improve the detection rate of a variety of digestive tract diseases in gastrointestinal examination, and serve clinical work better as a quality control system.

## Author Contributions

All authors contributed to the writing and editing of the manuscript and contributed to the article and approved the submitted version.

## Conflict of Interest

The authors declare that the research was conducted in the absence of any commercial or financial relationships that could be construed as a potential conflict of interest.

## References

[1] Parasa S Wallace M Bagci U Antonino M Berzin T Byrne M. Proceedings from the first global artificial intelligence in gastroenterology and endoscopy summit. Gastrointest Endosc. (2020) 92:938–45.e1. 10.1016/j.gie.2020.04.044 32343978

[2] Chahal D Byrne MF. A primer on artificial intelligence and its application to endoscopy. Gastrointest Endosc. (2020) 92:813–20.e4. 10.1016/j.gie.2020.04.074 32387497

[3] Topol EJ. High-performance medicine: the convergence of human and artificial intelligence. Nat Med. (2019) 25:44–56. 10.1038/s41591-018-0300-7 30617339

[4] Anirvan P Meher D Singh SP. Artificial intelligence in gastrointestinal endoscopy in a resource-constrained setting: a reality check. Euroasian J Hepatogastroenterol. (2020) 10:92–7. 10.5005/jp-journals-10018-1322 33511071 PMC7801886

[5] Ahmad OF Soares AS Mazomenos E Brandao P Vega R Seward E. Artificial intelligence and computer-aided diagnosis in colonoscopy: current evidence and future directions. Lancet Gastroenterol Hepatol. (2019) 4:71–80. 10.1016/S2468-1253(18)30282-6 30527583

[6] Chartrand G Cheng PM Vorontsov E Drozdzal M Turcotte S Pal CJ. Deep learning: a primer for radiologists. Radiographics. (2017) 37:2113–31. 10.1148/rg.2017170077 29131760

[7] Ruffle JK Farmer AD Aziz Q. Artificial intelligence-assisted gastroenterology- promises and pitfalls. Am J Gastroenterol. (2019) 114:422–8. 10.1038/s41395-018-0268-4 30315284

[8] Rey JF Lambert R Committee EQA. ESGE recommendations for quality control in gastrointestinal endoscopy: guidelines for image documentation in upper and lower GI endoscopy. Endoscopy. (2001) 33:901–3. 10.1055/s-2001-42537 11605605

[9] Takiyama H Ozawa T Ishihara S Fujishiro M Shichijo S Nomura S. Automatic anatomical classification of esophagogastroduodenoscopy images using deep convolutional neural networks. Sci Rep. (2018) 8:7497. 10.1038/s41598-018-25842-6 29760397 PMC5951793

[10] Wu L Zhang J Zhou W An P Shen L Liu J. Randomised controlled trial of WISENSE, a real-time quality improving system for monitoring blind spots during esophagogastroduodenoscopy. Gut. (2019) 68:2161–9. 10.1136/gutjnl-2018-317366 30858305 PMC6872441

[11] Chen D Wu L Li Y Zhang J Liu J Huang L. Comparing blind spots of unsedated ultrafine, sedated, and unsedated conventional gastroscopy with and without artificial intelligence: a prospective, single-blind, 3-parallel-group, randomized, single-center trial. Gastrointest Endosc. (2020) 91:332–9.e3. 10.1016/j.gie.2019.09.016 31541626

[12] Choi SJ Khan MA Choi HS Choo J Lee JM Kwon S. Development of artificial intelligence system for quality control of photo documentation in esophagogastroduodenoscopy. Surg Endosc. (2021). 10.1007/s00464-020-08236-6. [Epub ahead of print]. 33415420

[13] Abadir AP Ali MF Karnes W Samarasena JB. Artificial intelligence in gastrointestinal endoscopy. Clin Endosc. (2020) 53:132–41. 10.5946/ce.2020.038 32252506 PMC7137570

[14] Hassan C Wallace MB Sharma P Maselli R Craviotto V Spadaccini M. New artificial intelligence system: first validation study versus experienced endoscopists for colorectal polyp detection. Gut. (2020) 69:799–800. 10.1136/gutjnl-2019-319914 31615835

[15] Muto M Minashi K Yano T Saito Y Oda I Nonaka S. Early detection of superficial squamous cell carcinoma in the head and neck region and esophagus by narrow band imaging: a multicenter randomized controlled trial. J Clin Oncol. (2010) 28:1566–72. 10.1200/JCO.2009.25.4680 20177025 PMC2849774

[16] Ishihara R Takeuchi Y Chatani R Kidu T Inoue T Hanaoka N. Prospective evaluation of narrow-band imaging endoscopy for screening of esophageal squamous mucosal high-grade neoplasia in experienced and less experienced endoscopists. Dis Esophagus. (2010) 23:480–6. 10.1111/j.1442-2050.2009.01039.x 20095991

[17] Reid BJ Blount PL Feng Z Levine DS. Optimizing endoscopic biopsy detection of early cancers in Barrett's high-grade dysplasia. Am J Gastroenterol. (2000) 95:3089–96. 10.1111/j.1572-0241.2000.03182.x 11095322

[18] Falk GW Rice TW Goldblum JR Richter JE. Jumbo biopsy forceps protocol still misses unsuspected cancer in Barrett's esophagus with high-grade dysplasia. Gastrointest Endosc. (1999) 49:170–6. 10.1016/S0016-5107(99)70482-7 9925694

[19] Sharma P Savides TJ Canto MI Corley DA Falk GW Goldblum JR. The American Society for Gastrointestinal Endoscopy PIVI (preservation and incorporation of valuable endoscopic innovations) on imaging in Barrett's esophagus. Gastrointest Endosc. (2012) 76:252–4. 10.1016/j.gie.2012.05.007 22817781

[20] de Groof AJ Struyvenberg MR van der Putten J van der Sommen F Fockens KN Curvers WL. Deep-learning system detects neoplasia in patients with Barrett's esophagus with higher accuracy than endoscopists in a multistep training and validation study with benchmarking. Gastroenterology. (2020) 158:915–29.e4. 10.1053/j.gastro.2019.11.030 31759929

[21] Shichijo S Nomura S Aoyama K Nishikawa Y Miura M Shinagawa T. Application of convolutional neural networks in the diagnosis of helicobacter pylori infection based on endoscopic images. EBioMedicine. (2017) 25:106–11. 10.1016/j.ebiom.2017.10.014 29056541 PMC5704071

[22] Gulati S Patel M Emmanuel A Haji A Hayee B Neumann H. The future of endoscopy: advances in endoscopic image innovations. Dig Endosc. (2020) 32:512–22. 10.1111/den.13481 31286574

[23] Mohammadian T Ganji L. The diagnostic tests for detection of helicobacter pylori infection. Monoclon Antib Immunodiagn Immunother. (2019) 38:1–7. 10.1089/mab.2018.0032 30648911

[24] Misawa M Kudo SE Mori Y Nakamura H Kataoka S Maeda Y. Characterization of colorectal lesions using a computer-aided diagnostic system for narrow-band imaging endocytoscopy. Gastroenterology. (2016) 150:1531–2.e3. 10.1053/j.gastro.2016.04.004 27072671

[25] Rath T Tontini GE Vieth M Nagel A Neurath MF Neumann H. *In vivo* real-time assessment of colorectal polyp histology using an optical biopsy forceps system based on laser-induced fluorescence spectroscopy. Endoscopy. (2016) 48:557–62. 10.1055/s-0042-102251 27009081

[26] Byrne MF Shahidi N Rex DK. Will computer-aided detection and diagnosis revolutionize colonoscopy? Gastroenterology. (2017) 153:1460–4.e1. 10.1053/j.gastro.2017.10.026 29100847

[27] Sinonquel P Eelbode T Bossuyt P Maes F Bisschops R. Artificial intelligence and its impact on quality improvement in upper and lower gastrointestinal endoscopy. Dig Endosc. (2021) 33:242–53. 10.1111/den.13888 33145847

[28] Nakagawa K Ishihara R Aoyama K Ohmori M Nakahira H Matsuura N. Classification for invasion depth of esophageal squamous cell carcinoma using a deep neural network compared with experienced endoscopists. Gastrointest Endosc. (2019) 90:407–14. 10.1016/j.gie.2019.04.245 31077698

[29] Tokai Y Yoshio T Aoyama K Horie Y Yoshimizu S Horiuchi Y. Application of artificial intelligence using convolutional neural networks in determining the invasion depth of esophageal squamous cell carcinoma. Esophagus. (2020) 17:250–6. 10.1007/s10388-020-00716-x 31980977

[30] Kubota K Kuroda J Yoshida M Ohta K Kitajima M. Medical image analysis: computer-aided diagnosis of gastric cancer invasion on endoscopic images. Surg Endosc. (2012) 26:1485–9. 10.1007/s00464-011-2036-z 22083334

[31] Zhu Y Wang QC Xu MD Zhang Z Cheng J Zhong YS. Application of convolutional neural network in the diagnosis of the invasion depth of gastric cancer based on conventional endoscopy. Gastrointest Endosc. (2019) 89:806–15 e1. 10.1016/j.gie.2018.11.011 30452913

[32] Kanesaka T Lee TC Uedo N Lin KP Chen HZ Lee JY. Computer-aided diagnosis for identifying and delineating early gastric cancers in magnifying narrow-band imaging. Gastrointest Endosc. (2018) 87:1339–44. 10.1016/j.gie.2017.11.029 29225083

[33] Miyaki R Yoshida S Tanaka S Kominami Y Sanomura Y Matsuo T. A computer system to be used with laser-based endoscopy for quantitative diagnosis of early gastric cancer. J Clin Gastroenterol. (2015) 49:108–15. 10.1097/MCG.0000000000000104 24583752

[34] Wang Y Tavanapong W Wong J Oh JH de Groen PC. Polyp-alert: near real-time feedback during colonoscopy. Comput Methods Programs Biomed. (2015) 120:164–79. 10.1016/j.cmpb.2015.04.002 25952076

[35] Requa J Dao T Ninh A Karnes W. Can a convolutional neural network solve the polyp size dilemma? Category award (colorectal cancer prevention) presidential poster award. Am J Gastroenterol. (2018) 113:S158. 10.14309/00000434-201810001-00282

[36] Byrne MF Chapados N Soudan F Oertel C Linares Perez M Kelly R. Real-time differentiation of adenomatous and hyperplastic diminutive colorectal polyps during analysis of unaltered videos of standard colonoscopy using a deep learning model. Gut. (2019) 68:94–100. 10.1136/gutjnl-2017-314547 29066576 PMC6839831

[37] Ito N Kawahira H Nakashima H Uesato M Miyauchi H Matsubara H. Endoscopic diagnostic support system for cT1b colorectal cancer using deep learning. Oncology. (2019) 96:44–50. 10.1159/000491636 30130758

[38] Chen PJ Lin MC Lai MJ Lin JC Lu HH Tseng VS. Accurate classification of diminutive colorectal polyps using computer-aided analysis. Gastroenterology. (2018) 154:568–75. 10.1053/j.gastro.2017.10.010 29042219

[39] Thakkar S Carleton NM Rao B Syed A. Use of artificial intelligence-based analytics from live colonoscopies to optimize the quality of the colonoscopy examination in real time: proof of concept. Gastroenterology. (2020) 158:1219–21 e2. 10.1053/j.gastro.2019.12.035 31945357 PMC7103545

[40] Zhou J Wu L Wan X Shen L Liu J Zhang J. A novel artificial intelligence system for the assessment of bowel preparation (with video). Gastrointest Endosc. (2020) 91:428–35.e2. 10.1016/j.gie.2019.11.026 31783029

[41] Filip D Gao X Angulo-Rodriguez L Mintchev MP Devlin SM Rostom A. Colometer: a real-time quality feedback system for screening colonoscopy. World J Gastroenterol. (2012) 18:4270–7. 10.3748/wjg.v18.i32.4270 22969189 PMC3436041

[42] Gong D Wu L Zhang J Mu G Shen L Liu J. Detection of colorectal adenomas with a real-time computer-aided system (ENDOANGEL): a randomised controlled study. Lancet Gastroenterol Hepatol. (2020) 5:352–61. 10.1016/S2468-1253(19)30413-3 31981518

[43] Arribas J Antonelli G Frazzoni L Fuccio L Ebigbo A van der Sommen F. Standalone performance of artificial intelligence for upper GI neoplasia: a meta-analysis. Gut. (2020). 10.1136/gutjnl-2020-321922. [Epub ahead of print]. 33127833

[44] Frazzoni L Arribas J Antonelli G Libanio D Ebigbo A van der Sommen F. Endoscopists' diagnostic accuracy in detecting upper gastrointestinal neoplasia in the framework of artificial intelligence studies. Endoscopy. (2021). 10.1055/a-1500-3730. [Epub ahead of print]. 33951743

